# Influence of a Concurrent Exercise Training Intervention during Pregnancy on Maternal and Arterial and Venous Cord Serum Cytokines: The GESTAFIT Project

**DOI:** 10.3390/jcm8111862

**Published:** 2019-11-03

**Authors:** Pedro Acosta-Manzano, Irene Coll-Risco, Mireille N. M. Van Poppel, Víctor Segura-Jiménez, Pedro Femia, Lidia Romero-Gallardo, Milkana Borges-Cosic, Javier Díaz-Castro, Jorge Moreno-Fernández, Julio J. Ochoa-Herrera, Virginia A. Aparicio

**Affiliations:** 1 PA-HELP “Physical Activity for Health Promotion, CTS-1018” Research group, Department of Physical Education and Sport, Faculty of Sport Sciences. University of Granada, 18011 Granada, Spain; pacostamanzano@gmail.com (P.A.-M.); lidiaromerogallardo@gmail.com (L.R.-G.); milkana@ugr.es (M.B.-C.); 2 Sport and Health Research Centre, University of Granada, 18007 Granada, Spain; virginiaparicio@ugr.es; 3 Department of Physiology, Faculty of Pharmacy, Institute of Nutrition and Food Technology, University of Granada, 18011 Granada, Spain; javierdc@ugr.es (J.D.-C.); jorgemf@ugr.es (J.M.-F.); jjoh@ugr.es (J.J.O.-H.); 4 Institute of Sport Science, University of Graz, Graz 8010, Austria; mireille.van-poppel@uni-graz.at; 5 Department of Physical Education, Faculty of Education Sciences, University of Cádiz, 11519 Cádiz, Spain; victor.segura@uca.es; 6 Biomedical Research and Innovation Institute of Cádiz (INiBICA) Research Unit, Puerta del Mar University Hospital University of Cádiz, 11009 Cádiz, Spain; 7 Unit of Biostatistics, Department of Statistics, Faculty of Medicine, University of Granada, 18016 Granada, Spain; pfemia@ugr.es

**Keywords:** immune, cytokines, interleukin 1B, interleukin 6, interferon-gamma, tumor necrosis factor

## Abstract

The aim of the present study was to analyze the influence of a supervised concurrent exercise-training program, from the 17th gestational week until delivery, on cytokines in maternal (at 17th and 35th gestational week, and at delivery) and arterial and venous cord serum. Fifty-eight Caucasian pregnant women (age: 33.5 ± 4.7 years old, body mass index: 23.6 ± 4.1kg/m^2^) from the GESTAFIT Project (exercise (*n* = 37) and control (*n* = 21) groups) participated in this quasi-experimental study (per-protocol basis). The exercise group followed a 60-min 3 days/week concurrent (aerobic-resistance) exercise-training from the 17th gestational week to delivery. Maternal and arterial and venous cord serum cytokines (fractalkine, interleukin (IL)–1β, IL-6, IL-8, IL-10, interferon (IFN)–γ, and tumor necrosis factor (TNF)–α) were assessed using Luminex xMAP technology. In maternal serum (after adjusting for the baseline values of cytokines), the exercise group decreased TNF-α (from baseline to 35th week, *p* = 0.02), and increased less IL-1β (from baseline to delivery, *p* = 0.03) concentrations than controls. When adjusting for other potential confounders, these differences became non-significant. In cord blood, the exercise group showed reduced arterial IL-6 and venous TNF-α (*p* = 0.03 and *p* = 0.001, respectively) and higher concentrations of arterial IL-1β (*p* = 0.03) compared to controls. The application of concurrent exercise-training programs could be a strategy to modulate immune responses in pregnant women and their fetuses. However, future research is needed to better understand the origin and clearance of these cytokines, their role in the maternal-placental-fetus crosstalk, and the influence of exercise interventions on them.

## 1. Introduction

Pregnancy is a critical period of women’s life characterized by different immunometabolic responses depending on the trimester of pregnancy [1,2,3]. The fluctuations in these inflammatory responses are essential for adequate maternofetal homeostasis and thus, for a healthy and in-term pregnancy [1,2,3]. The first trimester of pregnancy is a highly anabolic phase accompanied by a pro-inflammatory state. This phase is followed by an anti-inflammatory state during the growth of the fetus, which finally turns into a pro-inflammatory state during late pregnancy (preparation for parturition) [1,2]. Nonetheless, an exacerbation or dysregulation of pro and anti-inflammatory cytokines might lead to a higher risk of developing pregnancy complications [1,3,4,5,6,7].

Importantly, not only the maternal immune system but also the placenta and fetus are sources of cytokines during pregnancy and are continuously interacting between them to balance pro and anti-inflammatory states [1,2,3,5,6,8,9,10,11]. Unfortunately, the metabolism, origin, and clearance of the different cytokines and their role and contribution to the maternal-placental-fetus crosstalk are currently poorly understood [1,3,7,8,10,12]. Hence, it seems important to better explore this matter to facilitate the research of more adequate strategies aimed at preventing related disruptions.

In this regard, exercise might be a promising clinical tool to modulate inflammatory responses and prevent complicated pregnancies [13]. The emerging role of skeletal muscle as a primordial endocrine organ [14,15] and its characteristic interplay with other organs via muscle contraction-induced factors (myokines) [9,14,15,16] could partially explain the beneficial effects of acute exercise (stress-like response) and long-term exercise (chronic adaptive response) on immunometabolic health [14,15]. However, this issue remains currently unperceived in pregnancy, where these cytokines could play a key/relevant function at the maternal-fetal interface [13,17].

Thus far, the majority of exercise interventions during pregnancy have mainly focused on a few classical markers, such as maternal plasma C-reactive protein and leptin. However, no studies to date have analyzed the effect on the inflammatory profile (including relevant cytokines such as fractalkine, interleukin (IL)-1β, IL-6, IL-8, IL-10, interferon-gamma (IFN-γ), and tumor necrosis factor-alpha (TNF-alpha)) of healthy pregnant women (without metabolic dysregulations) during pregnancy. Hence, it is of clinical interest to determine if exercise could be a potential strategy to modulate inflammatory responses of pregnant women and their fetuses.

The aim of this study was to analyze the influence of a supervised concurrent exercise-training program from the 17th gestational week until delivery on inflammatory markers in venous maternal (at 17th and 35th gestational week, and at delivery) and arterial and venous cord serum.

## 2. Methods

### 2.1. Settings and Eligibility Criteria

The procedures, along with the inclusion-exclusion criteria (Table S1) of the GESTAFIT Project, are described elsewhere [18]. Three-hundred and eighty-four pregnant women attending their gynecologist at the 12th gestational week were informed about the project in the San Cecilio and Virgen de las Nieves University Hospitals (Granada, Spain). The recruitment was performed in three different waves. From all the initially interested participants, 159 women were finally recruited. All participants signed an informed consent after being individually informed about the study’s aims and procedures. The GESTAFIT project was approved by the Clinical Research Ethics Committee of Granada, Government of Andalusia, Spain (code: GESFIT-0448-N-15).

### 2.2. Sample Size

The required sample size was only determined for the primary outcomes (maternal weight gains and maternal/neonatal glycemic profile) of the GESTAFIT Project, and it was 52 pregnant women (26 per group) [18].

### 2.3. Randomization

Initially, this study was based on a randomized control trial design. However, the randomization design was finally broken because of some difficulties related to the complexity of maintaining women in the control group (avoiding high rates of withdrawal). These methodological and ethical barriers are frequent in antenatal exercise research, as previously argued [19]. Hence, it was decided to subsequently allocate pregnant women to the exercise/control group depending on their personal preference and convenience to attend the intervention sessions and the wave in which they were recruited.

### 2.4. General Procedure

Women were assessed twice (2 different days/assessment) during the study. Socio-demographic and clinical characteristics, dietary patterns, blood pressure, pre-gravid self-reported weight, body weight, and height were assessed on the first day of the 16th week (±2 weeks). Each participant was given an accelerometer to wear until the next appointment. One week later (17th week), blood samples of the mothers were collected by a nurse, and accelerometers were returned. In the 35th week, the same assessments (all but socio-demographic and clinical characteristics) were performed, with identical timing to the 16th week. The samples collected in the 35th gestational week were taken around 2–3 days after the last exercise session from the main training phase (before the final phase: preparation for active delivery).

On the delivery day, maternal, and arterial and venous umbilical cord blood samples were collected moments after the delivery, and obstetric and gynecological histories were collected through the “Pregnancy Health Document”. The person responsible for the training sessions was the only person informed of the allocation of participants to the training/control groups. The assessment procedures are further explained in Figure S1.

### 2.5. Intervention

#### 2.5.1. Exercise Group

Pregnant women in the exercise group participated in a concurrent -training program from the 17th week until delivery (3 days/week, 60 min/session) consisting of a combination of aerobic-resistance exercises of moderate-to-vigorous intensity. This exercise protocol was designed by an expert multidisciplinary team following the recommendations from the American College of Obstetrics and Gynecology [20]. The exercise group started with an informative and movement learning phase (3 sessions). In this initial phase, fundamental basic movement patterns were taught (hip and knee dominant, pull and push movements), and theoretical explanations were provided to the participants. Subsequently, the main exercise training phase lasted from the 18th until the 34th week and was focused on improving or maintaining physical fitness. The final phase during the last weeks of pregnancy was focused on the pelvic mobilization (preparation for the delivery). The detailed exercise sessions (Appendix A) and protocol, along with specific exercises, can be found elsewhere (Supplementary Materials section) [21]. The attendance at the training sessions was recorded (see Table S2). During this period, the research team also gave 7 talks to the participants aimed at providing them with basic pregnancy health-related information (detailed in Appendix B).

#### 2.5.2. Control Group

The participants in the control group were requested to continue with their daily activities. Because of ethical considerations, we also invited them to these 7 talks. We also used these meetings to maintain their commitment until the end of the program.

### 2.6. Outcome Measures

Gynecologists and midwives from the hospitals, and expert physiologists responsible for the assessment of these secondary outcomes, were blinded to the allocated treatment of the participants.

#### 2.6.1. Sociodemographic and Clinical Data

Women completed a self-reported questionnaire of sociodemographic (age, number of children, cohabitation, marital, and educational status, among others), reproductive history, and clinical (suffering or having suffered specific diseases, and drug consumption) data, and smoking and alcohol habits. All instructions needed to properly understand and complete the self-reported survey were given by the research team.

#### 2.6.2. Perinatal Outcomes

Data related to the type of delivery (natural, instrumental, or cesarean), its duration, number of abortions, and offspring sex were obtained from perinatal obstetric records (partogram).

#### 2.6.3. Weight Status

Pre-pregnancy weight was self-reported. The height and weight were assessed using a stadiometer (Seca 22, Hamburg, Germany) and scale (InBody R20; Biospace, Seoul, South Korea), respectively. Body mass index was calculated (weight(Kg)/height(m^2^)).

#### 2.6.4. Blood Pressure and Resting Heart Rate

The participants were asked to sit down and rest for at least 10 min before any blood pressure or heart rate test was performed. A blood pressure monitor (M6 monitor Omron, Hoolddorp, The Netherlands) was employed to assess systolic and diastolic blood pressure, and resting heart rate.

#### 2.6.5. Mediterranean Diet Score

The Mediterranean Diet Score (MDS) [22] is an index created to evaluate the degree of adherence to the Mediterranean dietary pattern. The consumption of each of those foods for further calculations was assessed with a food frequency questionnaire [23].

#### 2.6.6. Sedentary Time and Physical Activity

Sedentary time and physical activity were objectively assessed with triaxial accelerometry (ActiGraph GT3X+, Florida, US). Women wore the accelerometers around their waists over 9 consecutive days (minimum recording for inclusion in analyses: 7 days, ≥10 h/day) from the 16th week. Detailed information is provided in a previously published article [24].

#### 2.6.7. Blood Collection

In standardized fasting conditions (8–9 a.m.) at our research center, venous blood samples of all women (in a rested state) were extracted from the antecubital vein and collected in serum vacutainers. Immediately after the delivery, maternal (from the antecubital vein) and arterial and venous (from the umbilical cord) blood samples were also extracted and stored in serum tubes. Then, the samples were centrifuged to separate serum from formed elements. Subsequently, serum was frozen at −80 °C to avoid breaking the cold chain before the analysis in the laboratory. More detailed information is shown in Appendix C.

#### 2.6.8. Cytokines

Maternal and umbilical arterial and venous serum cytokines (fractalkine, IL-1β, IL-6, IL-8, IL-10, IFN-γ, and TNF-α) were measured using Luminex xMAP technology (detailed in Appendix C).

### 2.7. Statistical Analyses

As initially designed [21], the statistical analysis was conducted on a per-protocol basis. Only women who attended ≥75% of the exercise sessions and completed both baseline and follow-up assessments were included in the per-protocol analyses.

Descriptive statistics for continuous and categorical variables were performed to show the sociodemographic and clinical characteristics (Table 1), along with the cytokine concentrations of pregnant women (Table 2). To detect potential differences in the outcomes between the groups, the following statistical tests were performed: independent sample Student’s *t*-test (normal distribution, homoscedasticity), Welch´s test (normal distribution, heteroscedasticity) and Mann–Whitney U test (non-normal distribution) for continuous variables, and the Chi-square test for categorical variables.

Considering the asymmetry of some cord serum cytokines and the violation of some assumptions related to the generalization of the results, data preparation was employed for those models. Particularly, optimum Box-Cox transformations and a subtle variation of winsorizing were used to reduce the impact of potential sources of bias. Additional information, along with an “Outlier detection-management” section, is provided in Appendix D.

Subsequently, linear regression analyses were used to analyze the differences in cytokine concentrations between the control and exercise group at different time-points. In the multiple time-point analyses (Table 3), the changes in maternal serum cytokine concentrations (from baseline to 34th gestational week and delivery) were included in the regression analyses as dependent variables, and the group (control = 0, exercise = 1) as independent variable. In the single time-point analyses (Table 4), the arterial and venous cord serum cytokine concentrations were included in the regressions as dependent variables, and the group as an independent variable.

After considering relevant confounders suggested in previous literature, mostly confounders that were statistically-significant to the outcomes and influenced the relationship between the independent and dependent variables (i.e., meaningful change in the coefficient B of the independent variable when added), were included in the models. In the multiple point analyses in the 34th week, model 1 was adjusted for baseline values of the particular cytokine and adherence to the MDS; and model 2 was additionally adjusted for the relative percentage of daily total physical activity (PA) (total PA/accelerometer wearing time). In multiple point analyses for delivery, model 1 was adjusted for baseline values of the particular cytokine; and model 2 was additionally adjusted for parity status and weeks of gestation at delivery. In the single point time analyses, model 1 was adjusted for adherence to the MDS; and model 2 was additionally adjusted for parity status and gestational age at delivery. Since important variables (maternal age, BMI, tobacco, and daily total PA, among others) according to the literature showed a weak or no relationship with the outcomes, and we wanted to enhance the parsimony of the main models, these variables were only tested as additional confounders in a secondary sensitive analyses.

All the assumptions related to the generalization of the results (additivity, linearity, homoscedasticity, normal distribution of the residuals, no perfect multicollinearity, independence of errors, etc.) were met in the different analyses.

Multiple imputations were performed for those cases with missing data in specific outcomes. Subsequently, the aforementioned statistical analyses were conducted on an intention-to-treat basis to evaluate more realistically the effectiveness of this concurrent exercise-training program when applied to the clinical practice (Tables S3 and S4), according to the CONSORT guidelines.

The statistical analyses were conducted using SPSS 22.0 (IBM, Armonk, NY, USA). The statistical significance was set at *p* ≤ 0.05.

### 2.8. Patient and Public Involvement

The participants were not involved either in the design or conduct of this research. Pregnant women will be informed of their results through annual meetings and/or detailed reports suitable for a non-expert audience.

## 3. Results

**Figure 1:**
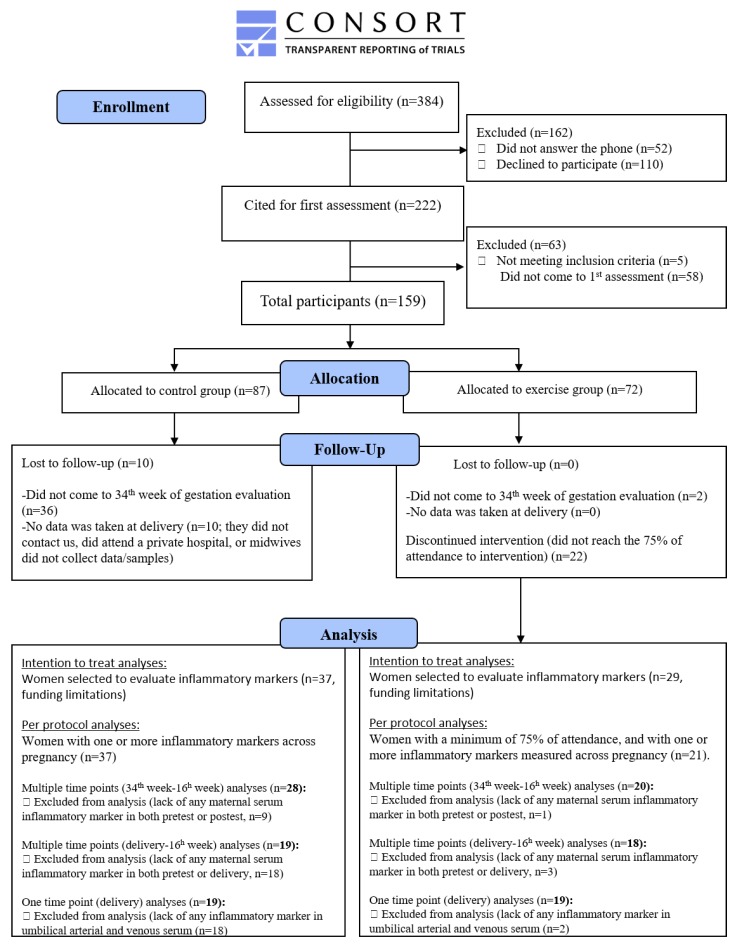
Flowchart of pregnant women through each stage of the study.

From all the initially interested participants (*n* = 384) between November 2015 and November 2017, the final study sample included in the per-protocol analyses (>75% of attendance) was 58 Caucasian southern Spanish pregnant women (age 33.5 ± 4.7 years old, BMI 23.6 ± 4.1 kg/m^2^). These women were divided into control (*n* = 37) and exercise (*n* = 21) groups. The follow-up for the last wave of participants was completed in April 2018. Further information about the allocation and analysis process, along with reasons for losses/exclusions, is provided in Figure 1 and Appendix E.

The sociodemographic and clinical characteristics of pregnant women are shown in Table 1. Baselines differences were found for the time spent undertaking light and total PA between the control and exercise group (*p* = 0.01 and *p* = 0.03, respectively). The mean exercise training adherence was approximately 84%.

The unadjusted differences in cytokine concentrations at the three time points are shown in Table 2. In maternal serum, differences were found in TNF–α (35th week) and IL-10 (delivery) concentrations (*p* = 0.03 and *p* = 005, respectively). Regarding the cord serum, the control group showed lower arterial serum IL-1β levels, and greater arterial serum IL-6 and venous TNF-α concentrations than the exercise group (*p*-values between 0.002–0.05).

The effects of the exercise intervention on maternal serum cytokines are shown in Table 3. In the regression analyses for changes from baseline to 35th week (model 1), the exercise group decreased 1.03 pg/mL (−1.89 to −0.18, *p* = 0.02) TNF-α concentrations compared to the control group. When analyzing changes from baseline to delivery (model 1), the exercise group was associated with a lower increase in IL-1β (−2.38 pg/mL, −4.53 to −0.22, *p* = 0.03), and a greater increase in IL-10 (9.40 pg/mL, 0.15 to 18.64, *p* = 0.05) compared to the control group. When additionally adjusting these analyses (model 2), the aforementioned differences for maternal TNF-α, IL-1β, and IL-10 became non-significant (*p* = 0.06, *p* = 0.07, and *p* = 0.08, respectively).

The effects of the exercise intervention on arterial and venous cord serum cytokines are shown in Table 4. In model 1, the exercise group was associated with higher arterial cord serum IL-1β (0.69 pg/mL, 0.30 to 0.08, *p* = 0.03) and lower arterial cord serum IL-6 (−0.79 pg/mL, −1.48 to −0.11, *p*-value = 0.02) compared to the control group. Regarding the venous cord cytokines (model 1), the exercise group was associated with lower TNF-α (−5.53 pg/mL, −8.47 to −2.60, *p* = 0.001) concentrations as compared with the control group. In model 2, the results remained similar.

When additionally adjusting the single/multiple point analyses for BMI, maternal age, tobacco, and daily total PA, the results did not change.

Because of the substantial percentage of missing data (average = 42.7%), multiple imputations were not possible for some outcomes. Intention-to-treat analyses have been added to Supplementary material (Tables S3 and S4) to be as transparent as possible. Considering that some authors do not recommend to perform imputations when more than 20% of cases are missing [25], we have not considered this data for the discussion.

The moderate-to-vigorous exercise intervention was shown to be safe. Non-adverse, potentially harmful, or unintended effects were observed in none of the groups.

## 4. Discussion

Under the framework of the GESTAFIT project, the present study shows for the first time the effects of a novel, well-designed and supervised individually-tailored concurrent exercise program [18] (based on the latest guidelines in pregnancy [13]) on maternal and arterial and venous cord serum cytokines. The main findings suggest that a concurrent exercise-training program might reduce arterial cord IL-6 and venous cord TNF-α concentrations. Unexpectedly, pregnant women from the exercise group showed higher concentrations of arterial cord IL-1β.

Until now, only two previous studies have presented similar results to those shown in the current study. Clapp et al. [26] conducted a weight-bearing exercise intervention from pregravid, but only focused on maternal serum TNF-α and leptin concentrations. Otherwise, Aparicio et al. [21] also showed similar results to those described above in breast milk.

Interleukin-6 is a pleiotropic well-known pro and anti-inflammatory cytokine [15,27] with relevant influence on the immunometabolic homeostatic responses during pregnancy [1,2]. Our results indicated that arterial cord serum IL-6 concentrations were reduced in the exercise compared to the control group, while non-significant changes were appreciated in maternal (at 34th week and delivery) and venous cord serum IL-6. Moran et al. [28] showed that dietary-PA counseling was not associated with either maternal or cord blood IL-6 concentrations. When comparing with the general population, some studies have suggested that exercise might reduce IL-6 expression in the skeletal muscle and plasma levels [14,15,29]. However, these results are inconclusive according to a recent systematic review [17]. Interestingly, we observed greater concentrations of arterial than venous cord serum IL-6 (Table S5), which might suggest that IL-6 synthesis during parturition is mainly induced by the fetus [8,12]. Hence, given that we found differences in arterial cord IL-6 (but not in maternal or cord venous IL-6) concentrations between the exercise and control groups, we hypothesize that exercise might modulate fetal synthesis of IL-6 and/or placental clearance during parturition [8,9,12]. However, in spite of the fact that induced pro-inflammatory responses are necessary for the normal physiological course of pregnancy and birth [1,2,3,8], exacerbated IL-6 concentrations have been related to pregnancy-related inflammatory complications [1,3]. Finally, yet importantly, it has been suggested that exercise-induced IL-6 might facilitate optimal fetal growth and neonatal body composition [9] by modulating the expression and activity of placental nutrient transporters. In light of the above, strategies targeting IL-6 regulation during pregnancy are of clinical relevance [1,3,27]. Within this context, this concurrent exercise intervention might facilitate IL-6 regulation, favoring an optimal pregnancy and fetal development, and the prevention of potential immunometabolic dysregulations.

Regarding TNF-α, Clapp et al. [26] have previously suggested that weight-bearing exercise attenuates the increase in TNF-α concentrations across pregnancy. Remarkably, our results also showed that maternal (at the 34th week) and venous cord TNF-α concentrations were lower in the exercise compared to the control group. These findings are particularly relevant if we consider that TNF-α is a major driver for metabolic disruptions (e.g., gestational diabetes mellitus) [7,15], pregnancy complications, and congenital disorders [1,3,6]. It is noteworthy that depending on the concentrations, receptor distribution and duration of its stimulation, this pro-inflammatory cytokine has an imperative-bimodal physiological-pathological role mediating beneficial/adverse effects on female reproduction and pregnancy [6]. Hence, it seems that exercise could be a promising target to modulate TNF-α concentrations at the maternal-fetal interface during pregnancy, which might help to prevent immunometabolic dysregulations and reproductive diseases. However, when interpreting this data, we should consider that most studies addressing the maternal-fetal/fetal-maternal transfer of TNF-α have been performed in deliveries without labors [10,12] (unlike our study), and TNF-α was under detection limit in the immunoassays of vaginal labors [8]. Moreover, we found comparable concentrations of cord arterial and venous TNF-α in our participants (Table S5). Therefore, it is not possible (or it is unsuitable) to conclude any exercise-induced underlying mechanism related to maternal, placental or fetal TNF-α.

Interleukin-1β is a pro-inflammatory cytokine highly involved in the pathogenesis of immunometabolic abnormalities, with a recently discovered role as a physiological-metabolic mediator [30]. During implantation and parturition, adequate induced-IL-1β responses are imperative in maternal-fetal communication to promote healthier pregnancies [1,3]. Surprisingly, our results showed that pregnant women from the exercise group presented higher concentrations of arterial cord IL-1β, with a similar but non-significant trend in venous cord IL-1β. By contrast, maternal IL-1β serum levels (at delivery) were slightly reduced in the exercise compared to the control group (evidence of statistical significance). Unfortunately, we could not find any previous study of pregnant women to compare these results. Notwithstanding, one similar study by Moran et al. [28] observed that dietary-PA counseling did not affect maternal or cord blood IL-1β. In the general population, evidence regarding the influence of exercise interventions on IL-1β is also scarce and inconclusive [17]. To explain the rise observed, we hypothesized that higher arterial cord IL-1β in the exercise group could be related to greater exercise-induced placental volume and vascularization [9], which in turn might lead to a higher proportional release of IL-1β into maternal-fetal circulation. However, we dismissed this hypothesis since: (i) we did not observe significant changes in either maternal or venous cord serum IL-1β, which should be logical assuming an IL-1β-interplay between the placenta and fetus; (ii) IL-1β was not detectable in previous uncomplicated in-term pregnancies in the absence of labor (suggesting the absence of any inflammatory fetal-placental response) [8], or was not able to cross the placenta (suggesting that the inflammatory response in fetal blood and amniotic fluid might be of fetal origin) [12]; and (iii) it is likely that unnoticed factors [11] (even if we have considered the most relevant confounders such as duration of delivery, type of delivery, etc.) related to parturition, which is an acute phase with huge influence on the immune system [3], might partially explain these differences.

Therefore, when interpreting these results, we should consider that labor might play a role in the acute elevation of some cytokines at term, and is not a simple process itself. Different mechanisms (fetal membrane cell senescence, circadian endocrine clocks, inflammatory, and mechanical factors, etc.), are coordinated in a sequential and progressive manner, to initiate and provoke the parturition [31]. Overall, senescence and fetal membrane injury (fetal tissue aging) stimulate inflammatory responses, which are diffused and propagated to other compartments (involvement of multiple organ systems such as the decidua and myometrium) [31]. This inflammatory overload transforms the quiescent myometrium into an active muscle, and leads to cervix dilation and membrane weakening, and thus to parturition. Of note, this idea of an induced pro-inflammatory status (e.g., elevated concentrations of IL-1β, IL-6, IL-8, TNF-α, etc.) at the maternal-fetal tissues for successful labors, has been supported by previous literature [1,2,3,31,32]. However, to date, our understanding of the pathways of parturition (specially in this novel field of research) is limited, and many of the labor phenotypes observed at term have not been fully characterized biologically. Thus, future research is warranted to confirm if these cytokines are induced in response to exercise during labor, and to further elucidate their role at the parturition.

To facilitate an easier interpretation of the findings, those associations which showed evidence of statistical significance have been discussed separately in the Appendix F. Therefore, getting conclusions from the comparison with previous studies in pregnant women is difficult since the scarce existing interventions are based on PA-dietary counseling (different kinds of metabolic stimuli-responses), and/or have not measured the cytokines included in this study. Moreover, the interpretation of the results is even more complicated given the discrepancies aggravated by: (i) assessments are usually performed in different gestational weeks; (ii) variable immunometabolic and weight statuses; (iii) different methodologies and tissues when analyzing cytokines; (iv) different single/multiple time point statistical analyses, and small statistical power; and (v) distinct type of deliveries, etc.

Some limitations need to be highlighted. Despite the initial random allocation of participants to the intervention or control group, this random component could not be kept ultimately because of some difficulties related to the complexity of maintaining women in the control group. Hence, selection bias might be present. However, the presence/absence of a randomized design itself is unlikely to be as determinant as the methodological quality of the study [33]. The results should be interpreted cautiously given the small sample size and considering that no correction for multiple comparisons was made (as usual in exploratory/secondary outcomes analyses). The lack of statistically significant differences might be related to reduced statistical power. Only interested women participated in the study. Some strengths also deserve to be mentioned: (i) this exercise program is a novel individually-tailored intervention designed by an expert multidisciplinary team based on the latest guidelines in pregnancy [13]; (ii) the exercise program was strictly supervised during the whole study and the attendance, intensity, and other related parameters were monitored periodically; (iii) this is the first time that the effect of exercise has been analyzed in all these cytokines (excepting TNF-α); (iv) the cytokines were measured at multiple time points (including delivery), and in both the artery and vein cord serum; and (v) and we have not only adjusted the analyses for baseline values but also for powerful confounders such as objectively measured PA (with such a tight criterion, 7 days of ≥10 h/day) and the MDS (among others).

## 5. Conclusions

This concurrent exercise program might be a complementary-alternative tool to modulate the immune status of pregnant women and their newborns. The development of similar exercise programs might avoid potential immunometabolic impairments and prevent pregnancy complications. However, further research focused on the origin and clearance of these cytokines, their role in the maternal-placental-fetus crosstalk, and the influence of exercise interventions on them (along with the underlying mechanisms), is warranted before reaching any certain conclusion.

## Figures and Tables

**Table 1:** Sociodemographic and clinical characteristics of pregnant women (*n* = 48).

	Total (*n* = 48)		Control (*n* = 28)		Intervention (*n* = 20)		*p*-Value
**Age (years)**	33.5	(4.7)	33.5	(4.7)	33.5	(4.8)	0.97
**Gestational age in the 1st assessment (weeks), 16th week**	16.8	(1.4)	16.9	(1.1)	16.6	(1.7)	0.68
**Gestational age in the 2nd assessment (weeks), 34th week**	33.0	(1.7)	32.1	(1.7)	31.6	(1.7)	0.28
**Gestational age at delivery (weeks)**	39.4	(1.4)	39.1	(1.6)	39.8	(1)	0.16
**Percentage of attendance**					83.9	(8.2)	
**Cohabitation, *n* (%)**							
Living alone	0	(0)	0	(0)	0	(0)	
Living with partner	48	(100)	28	(100)	20	(100)	
**Educational level, *n* (%)**							
Non-university degree	20	(41.7)	11	(39.3)	9	(45)	0.92
University degree	28	(58.3)	17	(60.7)	11	(55)	
**Professional status, *n* (%)**							
Work full/part time	31	(64.6)	21	(75)	10	(50)	0.14
Unemployed/Retired/Housekeeper	17	(35.4)	7	(25)	10	(50)	
**Parity status, *n* (%)**							
Primarious	28	(58.3)	14	(50)	14	(70)	0.28
Multiparious	20	(41.7)	14	(50)	6	(30)	
**Number of abortions**	0.5	(0.8)	0.5	(0.8)	0.5	(0.8)	0.64
**Type of deliver ^a^, *n* (%)**							
Spontaneous	27	(57.4)	16	(59.3)	11	(55)	0.20
Vacuum extraction	9	(19.1)	3	(11.1)	6	(30)	
Caesarean section	11	(23.4)	8	(29.6)	3	(15)	
**Offspring sex ^b^, *n* (%)**							
Male	24	(52.2)	13	(50)	11	(55)	0.97
Female	22	(47.8)	13	(50)	9	(45)	
**Body mass index pre-pregnancy ^e^ (kg/m^2^)**	23.2	(3.8)	22.8	(3.5)	24.0	(4.4)	0.32
**Body mass index (kg/m^2^), 16th week**	23.6	(4.1)	23.3	(3.5)	24.0	(4.9)	0.98
**Gestational weight gain from pre-pregnancy to 16th week ^e^ (kg)**	1.1	(2.8)	1.1	(3.2)	0.9	(2)	0.81
**Gestational weight gain from 16th to 34th week ^c^ (kg)**	9.5	(3.2)	10.1	(2.8)	8.7	(3.5)	0.234
**Cardiovascular function ^b^, 16th week**							
Systolic blood pressure (mmHg)	105.2	(8.8)	106.1	(9.1)	103.8	(8.4)	0.38
Diastolic blood pressure (mmHg)	61.9	(7.5)	61.5	(7.9)	62.4	(7)	0.70
Resting heart rate (bpm)	81.7	(10.8)	81.9	(10.7)	81.5	(11.3)	0.57
**Smoking during pregnancy (cigarettes per day), 16th week**	0.4	(1.6)	0.5	(2)	0.2	(0.9)	0.50
**Adherence to the Mediterranean Diet Score (0–55), 16th week**	29.1	(3.8)	29.3	(3.9)	28.8	(3.9)	0.73
**Sedentary lifestyle and physical activity ^d^, 16th week**							
Sedentary time (min/day)	503.9	(98.5)	486.0	(116.5)	526.4	(65.7)	0.19
Light PA (min/day)	392.5	(89.9)	420.8	(99.2)	356.7	(61.9)	**0.01**
Moderate-vigorous PA (min/day)	37.8	(23)	37.2	(26.1)	38.6	(19.0)	0.48
Bouted moderate-vigorous PA (min/week)	99.4	(120.1)	106.1	(141.6)	90.8	(89.1)	0.95
Total PA (min/day)	430.3	(93.0)	458.0	(99.1)	395.3	(73.0)	**0.03**
Average accelerometer wear time (min/day)	934.2	(53.5)	944.0	(61.5)	921.7	(39.3)	0.18
Relative percentage of daily sedentary time (%)	53.9	(9.7)	51.3	(10.7)	57.2	(7.4)	**0.05**
Relative percentage of total daily PA (%)	46.1	(9.7)	48.7	(10.7)	42.8	(7.4)	**0.05**

PA, physical activity. Continuous variables are presented as mean (standard deviation) and categorical variables as number (percentage). Superscripts in outcomes indicate lower sample size when considering all participants: ^a^ *n* = 47, ^b^ *n* = 46, ^c^ *n* = 45, ^d^ *n* = 43, ^e^ *n* = 41. P values were calculated using independent sample Student’s t-test (normal distribution, homoscedasticity), Welch´s test (normal distribution, heteroscedasticity) and Mann–Whitney U test (non-normal distribution) for continuous variables, and the Chi-square test for categorical variables.

**Table 2:** Cytokines concentrations at the three time points (*n* = 48).

Cytokines	17th Week of Gestation (*n* = 48)					35th Week of Gestation (*n* = 48)					Delivery (*n* = 38)				
	Control (*n* = 28)		Intervention (*n* = 20)		*p*-Value	Control (*n* = 28)		Intervention (*n* = 20)		*p*-Value	Control (*n* = 19)		Intervention (*n* = 19)		*p*-Value
Maternal Serum	Mean	SD	Mean	SD		Mean	SD	Mean	SD		Mean	SD	Mean	SD	
Fractalkine (pg/mL)	376.1	149.0	371.4	152.7	0.85	375.8	107.3	391.4	107.2	0.74	345.5	64.6	387.2	109.6	0.17
Interleukin 1 beta (pg/mL)	6.8	2.6	6.1	3.2	0.17	7.5	3.0	6.3	3.6	0.20	9.6	3.9	7.2	3.5	0.06
Interleukin 6 (pg/mL)	5.6	2.8	5.9	3.0	0.70	6.3	2.5	5.2	2.6	0.15	32.9	9.5	33.2	19.1	0.95
Interleukin 8 (pg/mL)	21.5	10.7	18.6	7.6	0.50	19.8	8.5	21.6	10.7	0.62	34.4	6.8	37.8	15.2	0.39
Interleukin 10 (pg/mL)	24.0	9.6	22.3	12.2	0.59	24.5	8.8	29.1	9.8	0.10	40.7	11.0	50.4	16.7	**0.05**
Interferon gamma (pg/mL)	23.5	11.0	22.6	12.8	0.57	22.9	11.5	18.4	9.3	0.15	18.8	6.6	15.5	7.0	0.14
Tumor necrosis factor alpha (pg/mL)	5.6	1.7	5.2	2.7	0.11	7.1	1.7	6.1	1.4	**0.03**	10.1	2.7	9.0	2.6	0.25
**Umbilical arterial serum ^a^**															
Fractalkine (pg/mL)											314.6	91.1	372.3	103.9	0.07
Interleukin 1 beta (pg/mL)											1.2	0.9	1.5	0.5	**0.03**
Interleukin 6 (pg/mL)											18.9	5.0	15.0	4.3	**0.02**
Interleukin 8 (pg/mL)											51.8	31.6	56.6	24.2	0.62
Interleukin 10 (pg/mL)											10.2	2.6	12.5	4.0	0.06
Interferon gamma (pg/mL)											3.2	1.5	2.6	1.1	0.15
Tumor necrosis factor-alpha (pg/mL)											15.8	3.5	14.3	2.9	0.17
**Umbilical venous serum**															
Fractalkine (pg/mL)											265.8	113.1	300.4	117.3	0.36
Interleukin 1 beta (pg/mL)											1.4	0.8	1.7	1.0	0.58
Interleukin 6 (pg/mL)											13.3	5.4	12.6	4.8	0.65
Interleukin 8 (pg/mL)											59.1	22.8	60.8	17.4	0.80
Interleukin 10 (pg/mL)											12.9	4.1	13.3	3.8	0.78
Interferon gamma (pg/mL)											2.4	1.0	2.8	1.4	0.41
Tumor necrosis factor-alpha (pg/mL)											19.0	5.4	13.9	3.9	**0.002**

SD, standard deviation. ^a^ indicate lower sample size of the control group (*n* = 15) in all the umbilical arterial serum inflammatory markers. *p* values were calculated using independent sample Student’s *t*-test (normal distribution, homoscedasticity), Welch´s test (normal distribution, heteroscedasticity) and Mann–Whitney U test (non-normal distribution) for continuous variables.

**Table 3:** Per-protocol analyses showing the effect of the concurrent exercise-training program on maternal serum cytokines (*n* = 48).

	Changes in Control Group		Changes in Exercise Group		Model Unadjusted				Model 1				Model 2				Adjusted *R*^2^ *
	Mean	SD	Mean	SD	B	SE	β	*p*-Value	B	SE	β	*p*-value	B	SE	β	*p*-Value	
**35th week-17th week** **(maternal serum, *n* = 48)**	**(*n* = 28)**		**(*n* = 20)**														
Fractalkine	−0.35	101.10	19.98	91.67	20.33	28.49	0.11	0.48	17.92	20.72	0.09	0.39	15.11	23.16	0.08	0.52	−0.011
Interleukin 1 beta	0.67	3.13	0.17	2.12	−0.50	0.81	−0.09	0.54	−0.79	0.76	−0.14	0.31	−1.17	0.82	−0.22	0.16	−0.013
Interleukin 6	0.74	3.27	−0.67	3.11	−1.41	0.94	−0.22	0.14	−1.19	0.73	−0.18	0.11	−1.11	0.78	−0.17	0.16	0.047
Interleukin 8	−1.68	9.48	3.05	7.40	4.73	2.54	0.26	0.07	3.38	2.23	0.19	0.14	4.51	2.60	0.25	0.09	0.070
Interleukin 10	0.55	13.74	6.80	8.88	6.25	3.50	0.25	0.08	4.66	2.47	0.19	0.07	4.39	2.66	0.18	0.11	0.044
Interferon gamma	−0.55	9.97	−4.21	9.65	−3.65	2.88	−0.18	0.21	−4.11	2.50	−0.21	0.11	−5.56	2.81	−0.27	0.06	0.013
Tumor necrosis factor alpha	1.51	2.29	0.86	2.52	−0.66	0.70	−0.14	0.35	−1.03	0.43	−0.22	**0.02**	−0.86	0.44	−0.19	0.06	0.019
**Delivery-17th week** **(maternal serum, *n* = 37)**	**(*n* = 19)**		**(*n* = 18)**														
Fractalkine	−3.22	69.74	4.36	101.34	7.57	28.47	0.05	0.79	24.09	20.9	0.14	0.26	26.66	22.00	0.16	0.23	−0.026
Interleukin 1 beta	3.24	2.86	0.86	3.78	−2.38	2.10	−0.34	**0.04**	−2.38	1.06	−0.34	**0.03**	−2.09	1.10	−0.30	0.07	0.093
Interleukin 6	26.91	9.86	27.06	18.93	0.15	4.92	0.01	0.98	0.23	4.97	0.01	0.96	−0.90	5.2	−0.03	0.86	−0.029
Interleukin 8	14.53	14.57	19.31	16.10	4.78	5.04	0.16	0.35	3.33	3.91	0.11	0.40	3.20	4.18	0.11	0.45	0.025
Interleukin 10	18.65	13.98	27.30	16.93	8.65	0.10	0.28	0.10	9.40	4.55	0.30	**0.05**	7.92	4.37	0.25	0.08	0.076
Interferon gamma	−2.50	9.08	−8.26	12.21	−5.76	3.53	−0.27	0.11	−3.97	2.03	−0.18	0.06	−3.31	2.09	−0.15	0.12	0.044
Tumor necrosis factor alpha	4.66	2.72	3.62	2.97	−1.04	0.94	−0.19	0.27	−1.03	085	−0.18	0.23	−0.83	0.87	−0.15	0.35	0.007

SD, standard deviation; B, unstandardized regression coefficient; SE, standard error; β, standardized regression coefficient. Per-protocol analyses were performed including only women who attended ≥75% of the exercise sessions. Linear regression analyses (enter method) were used to examine the differences in inflammatory markers between the control and exercise group. The within-group post-pre intervention changes (from the exercise training group minus the control group) on cytokine concentrations were included in the linear regression analyses as dependent variables, and the group (control = 0 and exercise = 1) as independent variable. When considering the “35th week-17th week” multiple point analyses, model 1 was adjusted for baseline values of the particular cytokine and adherence to the Mediterranean Diet score; and model 2 was additionally adjusted for the relative percentage of daily total physical activity (total physical activity/accelerometer wearing time). When considering the “delivery-17th week” multiple point analyses, model 1 was adjusted for baseline values of the particular cytokine; and model 2 was additionally adjusted for parity status and gestational age at birth. * The adjusted *R^2^* values shown are derived from the unadjusted model. All the assumptions related to the generalization of the results have been reasonably met, and non-transformations or data preparation of the outcomes were needed.

**Table 4:** Per-protocol analyses showing the effect of the concurrent exercise-training program on arterial and venous cord serum cytokines at delivery (*n* = 38).

	Model Unadjusted				Model 1				Model 2				Adjusted *R*^2 b^
	B	SE	β	*p*-value	B	SE	β	*p*-value	B	SE	β	*p*-Value	
**Umbilical arterial serum (delivery) ^a^**													
Fractalkine *	0.63	0.32	0.33	0.06	0.53	0.32	0.28	0.11	0.52	0.33	0.27	0.13	0.081
Interleukin 1 beta *	0.66	0.29	0.38	**0.03**	0.69	0.30	0.39	**0.03**	0.72	0.27	0.41	**0.01**	0.113
Interleukin 6 *	−0.83	0.32	−0.42	**0.02**	−0.79	0.33	−0.40	**0.02**	−0.80	0.34	−0.40	**0.03**	0.147
Interleukin 8	4.83	9.56	0.09	0.61	6.67	9.85	0.12	0.50	7.25	9.93	0.13	0.47	−0.023
Interleukin 10	2.32	1.20	0.32	0.06	2.14	1.24	0.30	0.10	2.14	1.23	0.30	0.09	0.076
Interferon gamma	−0.65	0.44	−0.25	0.15	−0.70	0.46	−0.27	0.14	−0.68	0.46	−0.27	0.15	0.034
Tumor necrosis factor alpha	−1.55	1.10	−0.24	0.17	−1.63	1.14	−0.25	0.17	−1.63	1.14	−0.26	0.16	0.029
**Umbilical venous serum (delivery)**													
Fractalkine	34.58	37.38	0.15	0.36	27.29	36.93	0.12	0.47	23.68	38.24	0.10	0.54	−0.004
Interleukin 1 beta *	0.21	0.32	0.11	0.53	0.18	0.33	0.10	0.58	0.17	0.33	0.09	0.62	0.011
Interleukin 6	−0.77	1.67	−0.08	0.65	−0.90	1.70	−0.09	0.60	−1.03	1.77	−0.10	0.56	0.006
Interleukin 8 *	0.20	0.32	0.11	0.53	0.22	0.33	0.11	0.50	0.16	0.33	0.08	0.64	−0.016
Interleukin 10	0.37	1.28	0.05	0.78	0.31	1.31	0.04	0.82	0.23	1.36	0.03	0.87	−0.025
Interferon gamma	0.34	0.41	0.14	0.41	0.24	0.39	0.10	0.54	0.29	0.41	0.12	0.49	−0.008
Tumor necrosis factor alpha	−5.07	1.54	−0.48	**0.002**	−5.53	1.45	−0.53	**0.001**	−5.21	1.45	−0.50	**0.001**	0.211

SD, standard deviation; B, unstandardized regression coefficient; SE, standard error; β, standardized regression coefficient. Per-protocol analyses were performed including only women who attended ≥75% of the exercise sessions. Linear regression analyses (enter method) were used to examine the differences in inflammatory markers between the control and exercise group. The umbilical arterial serum cytokine concentrations were included in the linear regression analyses as dependent variables, and the group (control = 0 and exercise = 1) as independent variable. Model 1 was adjusted for adherence to the Mediterranean Diet score and model 2 was additionally adjusted for parity status and gestational age at birth. * Optimum Box-Cox transformations and a subtle variation of winsorizing (convert back from a z-score: replacing extreme scores with a score equivalent to ±2.58 SDs from the mean) were performed on inflammatory markers. ^a^ indicate lower sample size of the control group (*n* = 15) in all umbilical arterial serum inflammatory markers. ^b^ the adjusted *R*^2^ values shown are derived from the unadjusted model (i.e., it assesses the individual influence of the exercise intervention without confounders). All the assumptions related to the generalization of the results have been reasonably met. After dealing with the problematic outcomes, the results remained similar (but with better and more symmetrical distribution of data) to the analyses without data preparation, excepting for the interleukin 1 beta which became statically significant.

## Supplementary Materials

The following are available online at https://www.mdpi.com/2077-0383/8/11/1862/s1, Table S1: Inclusion and exclusion criteria in the GESTAFIT project, Table S2: Exercise protocol of The Gestafit Project, Table S3: Intention to treat analyses showing the effect of the concurrent exercise-training program on maternal serum, Table S4: Intention to treat analyses showing the effect of the concurrent exercise-training program on arterial and venous cord serum cytokines at delivery, Table S5: Differences between arterial and venous cord serum inflammatory markers, Figure S1: Assessments conducted along the GESTAFIT Project.

## Appendix A. Exercise Sessions

Each exercise session included a 10-min warm-up period with walks, mobility and activation exercises. The main part of the first and last weekly sessions consisted of 40 min of exercises organized in two resistance circuits of 15 exercises (40” work/20” rest), alternating with cardiovascular blocks (concurrent training). The second session of the week was focused on aerobic training through dancing, proprioceptive and coordinative circuits, and interval walks. The intensity of the sessions was monitored with the 6–20 Borg scale [34]. Women were asked to train at their 12–16 perceived exertion rate during each session, until the final phase (preparation for the delivery after the 34th gestational week). Therefore, the intensity of all the sessions was individually adapted. The sessions finished with a 10-min cool-down period of stretching, breathing, relaxation and myofascial relief [21].

## Appendix B. Talks Provided to Pregnant Women

During the duration of the intervention, the research team gave seven lectures to pregnant women from both groups (exercise and control group) about: (1) the benefits of physical exercise for a better pregnancy, prevention and treatment of cardiovascular diseases and excessive weight gain; (2) ergonomic advice, exercises to perform at home, and strategies to increase their daily physical activity levels; (3) the benefits of the Mediterranean Diet and nutritional education during pregnancy; (4) how to avoid toxins and chemicals during pregnancy and breastfeeding; (5) pregnancy, postpartum and sex; (6) physical and mental preparation for the labor, what to expect; and (7) nutritional education towards breastfeeding. We also used these conferences to maintain control group commitment until the end of the program.

## Appendix C. Detailed Information about the Blood Samples Analyses

In standardized fasting conditions (8–9 a.m.) at our research center (at the 17th and 35th gestational week), venous blood samples (5mL) of all pregnant women were extracted from the antecubital vein and collected in serum vacutainers. Immediately after the delivery, maternal (from the antecubital vein) and arterial and venous (from the umbilical cord) blood samples were also extracted and stored in serum tubes. For the umbilical cord blood sampling, a trained midwife performed a double clamping of the umbilical cord in the first three minutes of the newborn’s life, with a minimum distance between both clamps of 10 cm. A 1 mL syringe was used for blood extraction. Then, the samples were centrifuged at 1750 rpm for 10 min at 4 °C in a refrigerated centrifuge (GS-6R Beckman, Fullerton, CA, USA) to separate serum from formed elements. Subsequently, serum was aliquoted and frozen at −80 °C to avoid breaking the cold chain before the analysis in the laboratory.

We employed Luminex xMAP technology based on MILLIPLEX MAP kits to assess the cytokine profile from the collected serum in pregnant women. Luminex xMAP technology (Millipore, Darmstadt, Germany) is a mix of three existing and proved technologies: the use of microspheres, flow cytometry, and laser technology, mixing digital signal processing and traditional chemistry immunoassay. Because of robust multiplexing, xMAP technology potentially delivers more data in less time than other bioassay products, with comparable results with enzyme-linked immunosorbent assay and microarray. The technology offers several other distinct advantages over traditional methods, such as speed and high throughput, versatility, flexibility, accuracy, and reproducibility. Particularly, for maternal pro-inflammatory and anti-inflammatory (fractalkine, interleukin-1β, interleukin-6, interleukin-8, interleukin-10, interferon-γ and tumor necrosis factor-α) determination, we used Human Sepsis Magnetic Bead Panel 3 Multiplex Assay (cat. No. HTH17MAG-14K). We prepared samples, reagents, and standards by following the manufacturer’s instructions. Equipment settings: 50 events per bead, gate settings: 8000–15,000, time out 60 s. The plate was read on LABScan 100 analyzer (Luminex Corporation, Texas, USA) with xPONENT software for data acquisition. The average values for each set of duplicate samples or standards were within 15% of the mean. We determined cytokine concentrations by comparing the mean of duplicate samples with the standard curve for each assay.

## Appendix D. Outlier Detection and Management

Nowadays, the presence of outliers is one of the most enduring and pervasive methodological changes in biomedical science research [35,36,37]. Worryingly, there is a lack of consensus about how addressing outliers (i.e., how defining, identifying, and handling them). Since the decisions that researchers make about this issue have important implications, we have included this section to promote transparency and the critical interpretation of the results, as previously recommended by several authors [35,36,37]. Although no specific guidelines exist about how to address outliers, several studies [35,36,37,38,39,40,41,42] (especially the one from Aguinis et al. [37]) have previously provided smart advice and recommendations to address them in the best possible way. Accordingly, the different steps to address outliers in the present study have been performed proceeding with the following recommendations. We have identified and handled outliers according to the basis for regressions, which are the main analyses involved in this study.

**Error Outliers:** During the assessments at the different time points, questionnaires and tests (where errors related to data recording, coding, manipulation, etc. were likely and easily observed) were checked to identify clear error outliers, and correct them immediately by asking women, repeating the corresponding test, etc.

When lacking, misleading or inaccurate data was identified posteriori (up to two weeks after the assessments), women were contacted to ensure the accuracy of these data points, or to correct these potential outliers (whenever appropriate for data) in the respective database. Singles construct techniques (box plots, descriptive statistics, percentage analyses, etc.) were performed to initially identify error outliers. Subsequently, we also employed multiple construct techniques to identify error outliers. Particularly, we identified outlyingness based on predictor (leverage values, Cook´s distance and standardized differences in beta) and residual scores (standardized residuals). When it was not possible/appropriate to correct these data points, and we were sure that their inaccuracy was related to human errors, device malfunction, miscalculations or similar circumstances (i.e., we had determined the cause of the identified outlying observation), these error outliers were removed from the respective database. Since these potential error outliers could have been caused by inherent variability in the data (in this case they would represent a legitimate part of the population), we were very prudent when identifying and handling them. We paid special attention to the reasoning behind the classification of data points as error outliers.

**Interesting Outliers:** After the application of this first filter to the database, there were several remaining interesting outliers which required additional analyses, in-depth. Thereby, we aimed at analyzing these interesting outliers with quantitative approaches (e.g., we tried to analyze differences in how predictors were able to predict high and low outlier scores). However, the number of outliers was minimum, and only appreciable in few outcomes, which prevented us from performing these analyses properly. As a consequence, we did not finally perform these analyses.

**Influential Outliers:** Since it is not legitimate to simply drop the remaining potential outliers from the analyses (they tend to increase error variance, reduce the power of statistical tests, etc.), nor plainly delete them without any basis (they could be part of the inherent variability of the distribution of data), we analyzed in-depth the influence of these outliers in the model. With the objective of checking their influence, we analyzed how the deletion of specific outliers could affect the change of the model fit (e.g., changes in R^2^; model fit outliers), and we paid attention to the Cook´s Distance and standardized DFBETAs to identify prediction outliers. If these remaining unusual cases were not finally identified as influential outliers, or they were identified but influenced the model slightly, these potential outliers were not handled (as observed in Table 3). In this case, these unusual data points were dropped in the analyses since they did not affect either the results or assumptions of the tests, and they could be caused by inherent variability in the data. By contrast, if these remaining unusual cases were confirmed as influential outliers which affect the model fit and parameter estimates (as appreciable in Table 4), those influential outliers were handled.

Considering the asymmetry of some cord serum cytokines and the violation of some assumptions related to the generalization of the results (which were partially caused by these influential outliers), data preparation was employed for those models. First, optimum Box-Cox transformations were employed to correct distributional problems, non-linearity, outliers, non-normality, etc. Subsequently, in those few outcomes where outliers were/remained influential, a subtle variation of winsorizing (convert back from a z-score: replacing extreme scores (z > 2.58/3.29) with a score equivalent to ±2.58/3.29 standard deviations from the mean) was employed to handle these outliers. After dealing with the problematic outcomes, the results remained similar (but with a better and more symmetrical distribution of data) to the analyses without data preparation (i.e., without applying Box-Cox transformations or handling of outliers), except for the IL-1β, which became statically significant.

## Appendix E. Reasons for Losses and Exclusions during the Enrollment and Follow-Up

From the 159 women that participated in the study and were allocated to the control (*n* = 87) or intervention (*n* = 72) group, 10 controls dropped out of the study because of: moving to another city (*n* = 1), unwillingness to continue (*n* = 7) or unknown reasons (*n* = 2). In the control group, 36 women did not come to the evaluation (34th week) because of personal reasons. Data loss (*n* = 10) at delivery was related to women who did not contact us, attended private hospitals, or midwives who did not collect data/samples. Because of funding limitations, the inflammatory markers could only be analyzed in a subsample (*n* = 66). Hence, 37 and 29 women from the control and exercise group (respectively), were included the intention-to-treat analyses (Table S3). Of the 29 women in the exercise group, only 21 attended 75% of the sessions.

## Appendix F. Effect of the Concurrent Exercise-Training Program on Maternal and Arterial and Venous Cord Serum Cytokines (Non-Statistically SIGNIFICANT Associations, but Evidence of STATISTICAL Significance)

To facilitate an easier interpretation of the results, and provide a more complete and transparent description of the findings, we have decided to discuss this section separately. However, considering the magnitude of the effects (along with the confidence interval/standard error), caution must be paid in order not to over-interpret these results, and to avoid misleading conclusions.

Overall, a non-significant trend characterized by higher arterial IL-10 concentrations in pregnant women from the intervention group was noticed. When focusing on the changes in maternal serum cytokines from baseline to 34th week and delivery, exercise showed a non-significant but clinically meaningful [43] association with greater IL-8 and IL-10, and lower IFN-γ and TNF-α levels in maternal serum at the 34th week; and with lower maternal IL-1β and higher maternal IL-10 at delivery.

Fractalkine (CX3CL1) is a chemokine with an important role in the fetal-placental vasculature development [44] and microglial cell migration-activation [45]. When considering its role as a myokine [14], previous studies have suggested that physical exercise is not related to either fractalkine mRNA nor to protein concentrations in the general population, which is in agreement with our results [14,16,46]. However, these findings cannot be directly compared as there are no previous similar studies on fractalkine in pregnant women.

Besides its pro-inflammatory role [1,3], muscle-derived IL-8 (CXCL8) can also act as an angiogenic factor during early and late gestation [15]. The slightly increased (non-significant statistical trend) maternal serum IL-8 concentrations observed in the exercise group (at the 34th week; *p* = 0.07) might be suggestive of muscle IL-8-induced angiogenesis and placenta vascularization [9,15]. Moran et al. [28] reported that their dietary-physical activity counseling intervention was not associated with either maternal (at 28th and 36th weeks) nor cord blood IL-8 concentrations.

One of the most anti-inflammatory immunosuppressive cytokines during pregnancy is IL-10 (e.g., inhibition of IL-1, IL-6, and TNF-α), [4] which plays a primordial role at the maternal-fetal interface [1,4]. Hence, the observed non-significant (statistical trend; *p* ≤ 0.1) positive associations in our study might suggest an exercise-induced IL-10 role. However, these results do not allow us to ascertain that conclusion. By comparison, Moran et al. [28] did not observe significant changes in IL-10 concentrations with a physical activity counseling intervention.

Interferon-gamma is a pleiotropic pro-inflammatory cytokine characterized by powerful immunomodulatory effects on immune responses [1,5], and it plays a relevant role in the endometrial vasculature remodeling, angiogenesis, and maintenance of the decidua [1,3,5]. The higher decrease of IFN-γ observed at the 34th week and delivery in the exercise group (although non-significant), might be indicative of a small and irrelevant effect of exercise on IFN-γ during pregnancy, as previously suggested in the general population [47]. Likewise, Moran et al. [28] did not observe any significant change in either maternal nor cord plasma IFN-γ between a physical activity counseling intervention and the control group.
