# Supplementary material for: Influence of a Concurrent Exercise Training Intervention during Pregnancy on Maternal and Arterial and Venous Cord Serum Cytokines: The GESTAFIT Project
Source: J Clin Med. 2019 Nov 3;8(11):1862. doi: 10.3390/jcm8111862 (PMC6912691; doi:10.3390/jcm8111862)

SUPPLEMENTARY MATERIAL

Table S1. Inclusion and exclusion criteria in the GESTAFIT project.

Inclusion criteria
- Pregnant women aged 25-40 years old with a normal pregnancy course.
- Answering "no" to all questions on the PARmed-X for pregnancy.
- Being able to walk without assistance.
- Being able to read and write properly.
- Informed consent: Being capable and willing to provide written consent.
Exclusion criteria
- Having acute or terminal illness.
- Having malnutrition.
- Being unable to conduct tests for assessing physical fitness or exercise during pregnancy.
- Underweight
- Having pregnancy risk factors (such as hypertension, type 2 diabetes, etc.).
- Having a multiple pregnancy.
- Having chromosopathy or fetal malformations.
- Having uterine growth restriction.
- Having fetal death.
- Having upper or lower extremity fracture in the past 3 months.
- Suffering neuromuscular disease or presence of drugs affecting neuromuscular function.
- Being registered in another exercise program.
- Performing more than 300 minutes of at least moderate physical activity per week.
- Being unwilling either to complete the study requirements or to be randomized into the control or intervention group.

Table S2. Exercise protocol of The GESTAFIT Project.

**SESION STRUCTURE**	**CONTENT**										
**WARM-UP**<br>10 minutes	Joint mobility and different walk modalities										
	**Attendance (%)**	82.8	82.8	78.7	70.4	73.0	64.4	67.2	64.1	62.1	60.5
**CONDITIONING**<br>40 minutes	**Week**	**1**	**2-4**	**5-6**	**7-8**	**9-10**	**11-12**	**13-14**	**15-16**	**17-18**	**+ 19**
	**Gestational week**	**17**	**18-20**	**21-22**	**23-24**	**25-26**	**27-28**	**29-30**	**31-32**	**33-34**	**+34**
	**Intensity (RPE)**		12-13	12-13	13-14	13-14	14-15	14-15	15-16	15-16	
	**Monday**<br><br>**CIRCUIT**<br>(muscular and cardiovascular blocks)	Familiarization and acquisition of the basic ergonomic movement patterns	5 RE x 3<br>1min REST<br>1 AE 5′<br>1min REST<br>5RE x3<br>1AE 5′<br>1min REST	5 RE x 3<br>1min REST<br>1 AE 5′<br>1min REST<br>5RE x3<br>1AE 5′<br>1min REST	5 RE x 3<br>1min REST<br>1 AE 5′<br>1min REST<br>5RE x3<br>1AE 5′<br>1min REST	5 RE x 3<br>1min REST<br>1 AE 5′<br>1min REST<br>5RE x3<br>1AE 5′<br>1min REST	5 RE x 3<br>1min REST<br>1 AE 5′<br>1min REST<br>5RE x3<br>1AE 5′<br>1min REST	5 RE x 3<br>1min REST<br>1 AE 5′<br>1min REST<br>5RE x3<br>1AE 5′<br>1min REST	5 RE x 3<br>1min REST<br>1 AE 5′<br>1min REST<br>5RE x3<br>1AE 5′<br>1min REST	5 RE x 3<br>1min REST<br>1 AE 5′<br>1min REST<br>5RE x3<br>1AE 5′<br>1min REST	Pelvic movements + integration pattern. Real transfer to delivery moment
	**Wednesday**<br>(cardiovascular block)	Familiarization and acquisition of the basic ergonomic movement patterns	Choreographies and aerobic exercises								Pelvic movements + integration pattern. Real transfer to delivery moment
	**Friday**<br><br>**CIRCUIT**<br>(muscular and cardiovascular blocks)	Familiarization and acquisition of the basic ergonomic movement patterns	5 RE x 3<br>1min REST<br>1 AE 5′<br>1min REST<br>5RE x3<br>1AE 5′<br>1min REST	5 RE x 3<br>1min REST<br>1 AE 5′<br>1min REST<br>5RE x3<br>1AE 5′<br>1min REST	5 RE x 3<br>1min REST<br>1 AE 5′<br>1min REST<br>5RE x3<br>1AE 5′<br>1min REST	5 RE x 3<br>1min REST<br>1 AE 5′<br>1min REST<br>5RE x3<br>1AE 5′<br>1min REST	5 RE x 3<br>1min REST<br>1 AE 5′<br>1min REST<br>5RE x3<br>1AE 5′<br>1min REST	5 RE x 3<br>1min REST<br>1 AE 5′<br>1min REST<br>5RE x3<br>1AE 5′<br>1min REST	5 RE x 3<br>1min REST<br>1 AE 5′<br>1min REST<br>5RE x3<br>1AE 5′<br>1min REST	5 RE x 3<br>1min REST<br>1 AE 5′<br>1min REST<br>5RE x3<br>1AE 5′<br>1min REST	Pelvic movements + integration pattern. Real transfer to delivery moment
**COOL-DOWN**<br>10 minutes	Myofascial release, stretching and relaxation exercises										

RPE, rating of perceived exertion; RE, resistance exercise; AE, aerobic exercise; REST, resting. The load will be gradually and individualized increased for each participant to reach the intensity designed for each session. Attendance (%) refers to the average percentage of sessions attended by women from the exercise group in the present study.

Table S3. Intention to treat analyses showing the effect of the concurrent exercise-training program on maternal serum cytokines (n=53).

	Changes in control group		Changes in exercise group		Unadjusted model				Model 1				Model 2			
	**Mean**	**SD**	**Mean**	**SD**	**B**	**SE**	**β**	**p-value**	**B**	**SE**	**β**	**p-value**	**B**	**SE**	**β**	**p-value**
**35th week-17th week (maternal serum, n=53)**	**(n=28)**		**(n=25)**													
Fractalkine (pg/ml)	-0.35	101.10	16.70	83.54	17.05	25.66	0.09	0.51	14.06	18.71	0.08	0.46	8.88	21.00	0.05	0.68
Interleukin 1 beta (pg/ml)	0.67	3.13	0.16	1.99	-0.50	0.73	-0.10	0.50	-0.76	0.69	-0.15	0.28	-1.28	0.75	-0.25	0.09
Interleukin 6 (pg/ml)	0.74	3.27	-0.15	3.26	-0.89	0.90	-0.14	0.33	-0.84	0.71	-0.13	0.24	-0.82	0.78	-0.12	0.30
Interleukin 8 (pg/ml)	-1.68	9.48	3.57	7.11	5.25	2.33	0.30	0.03	3.63	2.06	0.21	0.08	4.02	2.38	0.23	0.10
Interleukin 10 (pg/ml)	0.55	13.74	8.15	10.47	7.60	3.39	0.30	0.03	5.58	2.56	0.22	0.03	5.60	2.84	0.21	0.06
Interferon gamma (pg/ml)	-0.55	9.97	-2.37	10.24	-1.81	2.78	-0.09	0.52	-2.71	2.45	-0.14	0.28	-3.91	2.80	-0.19	0.17
Tumor necrosis factor alpha (pg/ml)	1.51	2.29	1.02	2.32	-0.49	0.63	-0.11	0.44	-0.77	0.40	-0.17	0.06	-0.51	0.42	-0.12	0.23
**Delivery-17th week (maternal serum, n=43)**	**(n=19)**		**(n=24)**													
Fractalkine (pg/ml)	-3.22	69.74	13.63	97.14	16.84	26.47	0.10	0.53	29.95	19.40	0.18	0.131	30.76	20.46	0.18	0.14
Interleukin 1 beta (pg/ml)	3.24	2.86	1.35	3.54	-1.89	1.00	-0.28	0.07	-1.94	0.98	-0.29	0.06	-1.70	1.03	-0.25	0.11
Interleukin 6 (pg/ml)	26.91	9.86	29.22	18.82	2.31	4.77	0.08	0.63	2.15	4.80	0.07	0.66	0.91	4.99	0.03	0.86
Interleukin 8 (pg/ml)	14.53	14.57	19.80	14.85	5.27	4.52	0.18	0.25	2.98	3.59	0.10	0.41	2.72	3.83	0.09	0.48
Interleukin 10 (pg/ml)	18.65	13.98	28.52	15.20	9.87	4.51	0.32	0.03	9.69	4.00	0.32	0.02	8.12	3.95	0.27	0.05
Interferon gamma (pg/ml)	-2.50	9.08	-7.19	11.53	-4.69	3.23	-0.22	0.15	-3.75	1.96	-0.18	0.06	-3.20	2.03	-0.15	0.12
Tumor necrosis factor alpha (pg/ml)	4.66	2.72	3.86	2.87	-0.81	0.86	-0.14	0.36	-0.78	0.79	-0.14	0.33	-0.62	0.80	-0.11	0.44

SD, standard deviation; B, unstandardized regression coefficient; SE, standard error; β, standardized regression coefficient. Linear regression analyses (enter method) were used to examine the differences on inflammatory markers between the control and exercise group. The within-group post-pre intervention changes (from the exercise training group minus the control group) on cytokines concentrations were included in the linear regression analyses as dependent variables, and the group (control=0 and exercise=1) as independent variable. When considering the "35th week-17th week" multiple point analyses, the model 1 was adjusted for baseline values of the particular cytokine and adherence to the Mediterranean Diet score; and the model 2 was additionally adjusted for the relative percentage of daily total physical activity (total physical activity/accelerometer wearing time). When considering the "delivery-17th week" multiple point analyses, the model 1 was adjusted for baseline values of the particular cytokine; and the model 2 was additionally adjusted for parity status and gestational age at birth.

Table S4. Intention to treat analyses showing the effect of the concurrent exercise-training program on arterial and venous cord serum cytokines at delivery (n=44).

	**Unadjusted model**				**Model 1**				**Model 2**			
	**B**	**SE**	**β**	**p-value**	**B**	**SE**	**β**	**p-value**	**B**	**SE**	**β**	**p-value**
**Umbilical arterial serum (delivery)[a]**												
Fractalkine (pg/ml)*	0.78	0.31	0.38	**0.02**	0.69	0.31	0.34	**0.03**	0.69	0.32	0.34	**0.04**
Interleukin 1 beta (pg/ml)*	0.54	0.27	0.31	0.06	0.55	0.28	0.32	0.06	0.57	0.26	0.33	**0.04**
Interleukin 6 (pg/ml)*	-0.81	0.31	-0.40	**0.02**	-0.76	0.32	-0.37	**0.02**	-0.78	0.32	-0.38	**0.02**
Interleukin 8 (pg/ml)	5.80	9.03	0.11	0.53	6.80	9.30	0.13	0.47	7.63	9.34	0.14	0.42
Interleukin 10 (pg/ml)	2.41	1.11	0.34	**0.04**	2.23	1.14	0.32	0.06	2.21	1.14	0.31	0.06
Interferon gamma (pg/ml)	-0.69	0.42	-0.26	0.11	-0.71	0.43	-0.27	0.11	-0.71	0.44	-0.27	0.12
Tumor necrosis factor alpha (pg/ml)	-1.52	1.01	-0.24	0.14	-1.62	1.05	-0.26	0.13	-1.65	1.04	-0.26	0.12
**Umbilical venous serum (delivery)**												
Fractalkine (pg/ml)	5.80	35.22	0.03	0.87	0.94	35.18	0.00	0.98	-4.38	35.98	-0.02	0.90
Interleukin 1 beta (pg/ml)*	-1.11	1.61	-0.11	0.49	-1.32	1.61	-0.13	0.42	-1.32	1.68	-0.13	0.44
Interleukin 6 (pg/ml)	0.20	0.31	0.10	0.53	0.21	0.31	0.10	0.51	0.15	0.32	0.08	0.64
Interleukin 8 (pg/ml)*	0.62	1.22	0.08	0.61	0.64	1.24	0.08	0.61	0.42	1.27	0.05	0.74
Interleukin 10 (pg/ml)	0.17	0.30	0.08	0.59	0.16	0.31	0.08	0.61	0.12	0.32	0.06	0.70
Interferon gamma (pg/ml)	0.34	0.37	0.14	0.37	0.26	0.36	0.11	0.47	0.28	0.38	0.12	0.46
Tumor necrosis factor alpha (pg/ml)	-4.10	1.52	-0.38	**0.01**	-4.45	1.47	-0.42	**0.004**	-4.30	1.51	-0.40	0.007

SD, standard deviation; B, unstandardized regression coefficient; SE, standard error; β, standardized regression coefficient. Linear regression analyses (enter method) were used to examine the differences on inflammatory markers between the control and exercise group. The umbilical arterial serum cytokines concentrations were included in the linear regression analyses as dependent variables, and the group (control=0 and exercise=1) as independent variable. The model 1 was adjusted for adherence to the Mediterranean Diet score; and the model 2 was additionally adjusted for parity status and gestational age at birth. * Optimum Box-Cox transformations and a subtle variation of winsorizing (convert back from a z-score: replacing extreme scores with a score equivalent to ±2.58 SDs from the mean) were performed on inflammatory markers. [a] indicate lower sample size of the control group in all umbilical arterial serum inflammatory markers.

Table S5. Differences between arterial and venous cord serum cytokines (n=34).

	**All participants (n=34)**					**Control group (n=15)**					**Intervention group (n=19)**				
	**Mean difference (Artery-Vein)**					**Mean difference (Artery-Vein)**					**Mean difference (Artery-Vein)**				
	**Mean**	**SD**	**Mean Dif.**	**SE**	**p-value**	**Mean**	**SD**	**Mean Dif.**	**SE**	**p-value**	**Mean**	**SD**	**Mean Dif.**	**SE**	**p-value**
**Arterial fractalkine**	346.9	101.3	61.6	18.5	**0.002**	314.6	91.0	48.4	26.9	0.09	372.3	103.9	71.9	25.6	**0.01**
**Venous fractalkine**	285.3	117.1				266.2	118.1				300.3	117.2			
**Arterial interleukin-6**	16.8	4.9	3.8	0.8	**<0.001**	18.9	4.9	5.4	1.1	**<0.001**	15.0	4.2	2.4	1.1	**0.04**
**Venous interleukin-6**	13.0	5.3				13.4	5.9				12.5	4.8			
**Arterial interleukin-8**	54.5	27.4	-7.4	4.9	0.14	51.7	31.6	-11.5	9.2	0.23	56.6	24.1	-4.1	4.8	0.41
**Venous interleukin-8**	61.9	20.2				63.3	23.7				60.7	17.4			
**Arterial interleukin-10**	11.5	3.6	-1.5	0.7	**0.04**	10.1	2.5	-2.4	0.8	**0.015**	12.4	4.0	-0.8	1.0	0.46
**Venous interleukin-10**	13.0	3.6				12.6	3.5				13.3	3.7			
**Arterial interleukin-1beta**	1.4	0.7	-0.2	0.2	0.32	1.1	0.9	-0.2	0.2	0.41	1.5	0.4	-0.1	0.2	0.59
**Venous interleukin-1beta**	1.6	0.9				1.4	0.8				1.6	1.0			
**Arterial interferon gamma**	2.9	1.3	0.2	0.2	0.31	3.2	1.4	.7	0.3	**0.05**	2.5	1.0	-0.1	0.2	0.50
**Venous interferon gamma**	2.6	1.3				2.4	1.0				2.7	1.4			
**Arterial TNF-alpha**	15.0	3.2	-1.0	0.8	0.22	15.8	3.5	-2.7	1.4	0.07	14.2	2.90	0.3	0.8	0.71
**Venous TNF-alpha**	16.0	4.8				18.5	4.5				13.9	3.91			

Dif., difference; SD, standard deviation; SE, standard error; TNF, tumor necrosis factor alpha.

Figure S1. Assessments conducted along the GESTAFIT Project.

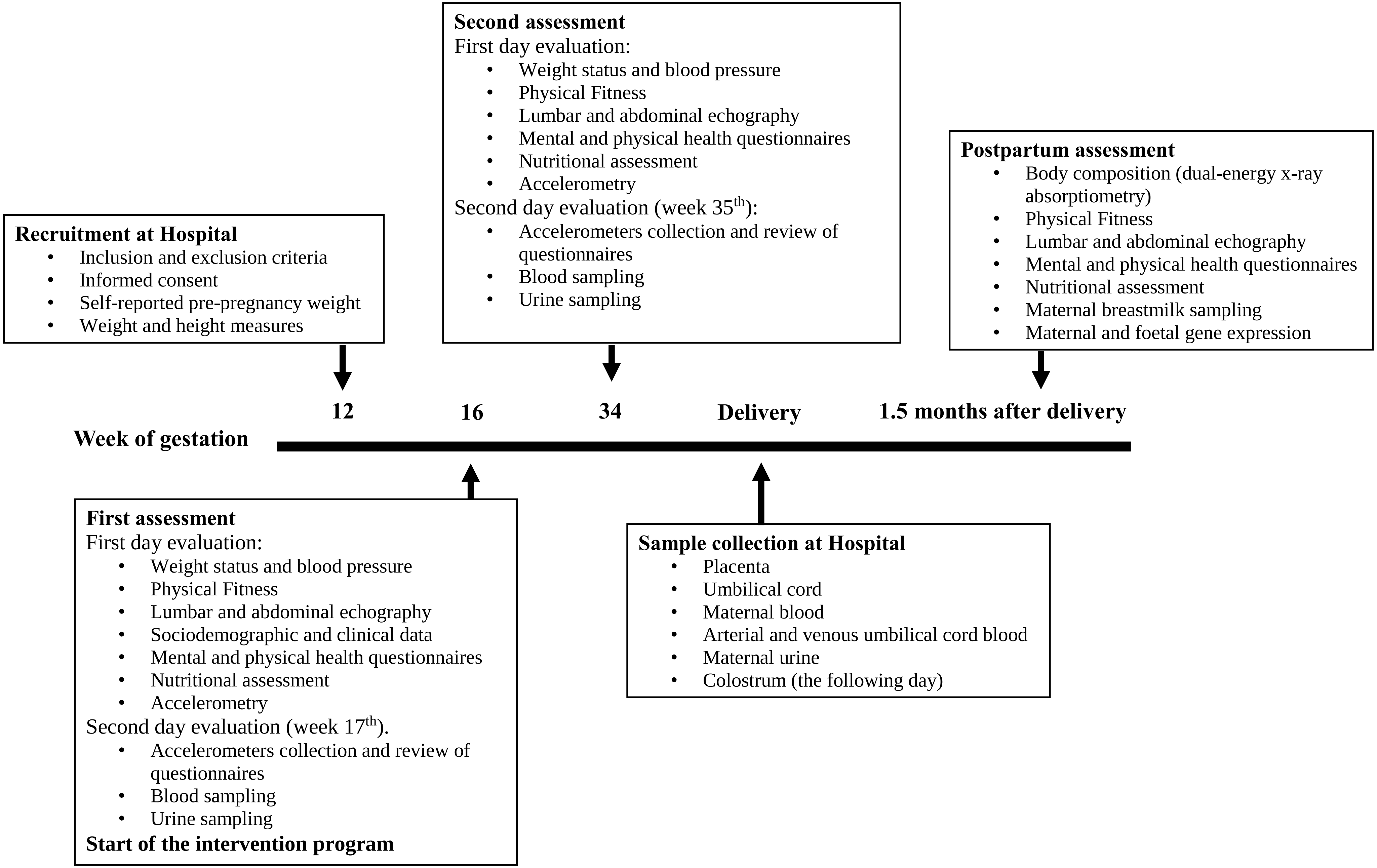

Supplement: Supplementary file 1 [file jcm-08-01862-s001.pdf]
